# How novice and expert anaesthetists understand expertise in anaesthesia: a qualitative study

**DOI:** 10.1186/s12909-020-02180-8

**Published:** 2020-08-12

**Authors:** Michael St.Pierre, James M. Nyce

**Affiliations:** 1 Anästhesiologische Klinik, Universitätsklinikum Erlange, Krankenhausstrasse 12, Erlangen, Germany; 2 Department of Anthropology, Ball State University, Muncie, IN USA

**Keywords:** Decision making, Expertise, Tacit knowledge, Qualitative research, Professionalism, Mentoring

## Abstract

**Background:**

The development of expertise in anaesthesia requires personal contact between a mentor and a learner. Because mentors often are experienced clinicians, they may find it difficult to understand the challenges novices face during their first months of clinical practice. As a result, novices’ perspectives may be an important source of pedagogical information for the expert. The aim of this study was to explore novice and expert anaesthetists understanding of expertise in anaesthesia using qualitative methods.

**Methods:**

Semi-structured interviews were conducted with 9 novice and 9 expert anaesthetists from a German University Hospital. Novices were included if they had between 3 and 6 months of clinical experience and experts were determined by peer assessment. Interviews were intended to answer the following research questions: What do novices think expertise entails and what do they think they will need to become an expert? What do experts think made them the expert person and how did that happen? How do both groups value evidence-based standards and how do they negotiate following written guidance with following one’s experience?

**Results:**

The clinical experience in both groups differed significantly (novices: 4.3 mean months vs. experts: 26.7 mean years; *p* < 0.001). Novices struggled with translating theoretical knowledge into action and found it difficult to talk about expertise. Experts no longer seem to remember being challenged as novice by the complexity of routine tasks. Both groups shared the understanding that the development of expertise was a socially embedded process. Novices assumed that written procedures were specific enough to address every clinical contingency whereas experts stated that rules and standards were essentially underspecified. For novices the challenge was less to familiarise oneself with written standards than to learn the unwritten, quasi-normative rules of their supervising consultant(s). Novices conceptualized decision making as a rational, linear process whereas experts added to this understanding of tacit knowledge and intuitive decision making.

**Conclusions:**

Major qualitative differences between a novice and an expert anaesthetist’s understanding of expertise can create challenges during the first months of clinical training. Experts should be aware of the problems novices may have with negotiating evidence-based standards and quasi-normative rules.

## Background

Experts are consensually defined as those who have been recognized within their profession as having the necessary skills and abilities to perform at the highest level [1]. A wealth of research on the idea of expertise has resulted in a list of features of expertise that seem to be found across many domains in art, science, and medicine [2–5]. In a nutshell, experts operate upon knowledge structures that are distincly different in its organization as well as in its extent from those of novices. Experts cognitively organize the perceptually available information in their working environment into larger meaningful patterns and can perceive at a more principled, functional, and abstract level the ‘deep structure’ of a problem or a situation. As a result of the very domain-specific acquired patterns and associated actions, experts have learned to retrieve relevant domain knowledge and strategies quickly with minimal cognitive effort and solve problems with little error.

The nature of expertise has been studied in two ways [6]: First, in an ‘absolute approach’ with a focus on truly exceptional individuals on the assumption that these individuals somehow have fundamental different qualities and understanding of their cognition and behavior. Second, in a ‘relative approach’ which assumes that there exists a continuum of capability between novice and expert and that expertise is a level of proficiency that novices can often achieve. In this perspective, expertise is created and maintained through collaborative and social processes, as well as through the perceptual and cognitive processes of the individual [7]. The literature on expertise provides different models that describe this path from novice to expert and identify characteristics and development activities at each stage. By passing through stages of qualitatively different perceptions of a task or a problem, expertise can be achieved [5]. In healthcare, the preferred developmental model thus far is that of Hubert and Stuart Dreyfus with its novice-to-master assessment rubric [8, 9].

Under the assumption that expertise is a level of proficiency that novices can achieve, expertise research has tried to develop instructional methods to support clinical teachers in helping their students to develop the types of knowledge representations, ways of thinking, and social practices that lay the foundations for the development of expertise. It is widely accepted that educational practices play an important role in creating (or inhibiting) the preconditions for expertise [10, 11].

As far as the professional development in anaesthesia is concerned, the core process in the transition from a novice anaesthetist to an expert practitioner appears to be in the integration and reconciliation of different types and streams of knowledge relevant to work in the operating room: factual knowledge that is presented in a structured and logical order, evidence-based guidelines from professional societies with their prescriptive stance towards decision making and action, and personal experience resulting in the subjective interpretation of knowledge.

The acquisition of these strategies and the successful transformation of explicit knowledge to tacit knowledge in anaesthesia seems to require direct personal contact between a mentor, or series of mentors, and a learner for transmission to take place [12]. Because mentors often are experienced clinicians, their teaching agenda will reflect the expert’s perspective and address aspects a novice has to acquire to be successful. However, recent developments conceptualise postgraduate training as educational alliance in which the trainees’ perspectives also play a pivotal role [13, 14]. As a result, it might be helpful for the expert supervisor to understand and build on novices’ perspectives on the development of expertise. Their viewpoint may represent an important source of pedagogical information for the clinical teacher as it can help to identify commonalities and differences in the understanding of novices and experts. Currently, our understanding of expertise in anaesthesia is solely based on interviews with study groups where participants had a wide range of clinical experience, ranging from 2 to 27 years [12, 15–19]. This research might help fill a gap in our understanding of expertise development as it adds the perspective of trainee anaesthetists in their first months of postgraduate medical training. Although it is not surprising to find differences between trainees and experts, it is not possible yet to say where junior and expert anaesthetists share common perceptions about expertise in clinical practice and where they differ. By interviewing novice and expert anesthesiologists of a single department at a German university hospital, this interview study was intended to answer the following research questions: What do novices think ‘expertise’ entails? What do they think they will need to become an expert? What do experts believe made them the expert person they are? How did that happen? What do they think novices should learn, see, and do in order to get there? How do novices and experts value standards and guidelines and how do they negotiate written guidance with following one’s experience and expertise?

## Methods

Prior to conducting the study, approval by the local ethics committee of the Friedrich-Alexander-Universität Erlangen-Nürnberg was obtained (reference number: 189_18). A qualitative research approach was used to compare novices’ and experts’ understanding of expertise in anaesthesia. The approach to qualitative research differs from that of quantitative research in that its primary goal is directed towards exploring human experience within a particular context rather than towards determining cause and effect or predicting and testing certain hypotheses. The qualitative research approach is particularly relevant for understanding the perspectives of the participants as collected in individual interviews.

### Participant selection

As statistical representativeness is not necessarily sought in qualitative research, the sampling strategy was determined by the purpose and duration of the research project. Anaesthesia trainees were included in the novice-group if the following criteria were met: a) no prior work experience in anaesthesia besides clinical rotations during medical school, b) between 3 to 6 months of clinical experience at our department at the time of the interview. The time frame was chosen to ensure that interviewees no longer were medical students and had gained a limited amount of clinical experience but at the same time were still at the beginning of their professional career. Based on numbers from previous years of newly employed trainees at the first author’s department it was estimated that 7 to 10 new junior doctors would fulfill these criteria during the data collection period April and October 2019. The investigator (M.StP) approached every novice face-to-face, explained the interview study and asked for their participation. Experts were selected by combining a) the certification level of a registered anaesthetist with b) peer vote from the remaining non-certified anaesthetists and anaesthetic nurses at the department. All non-registered physicians as well as all anaesthetic nurses received a list with the names of all registered anaesthetists (excluding the first author) and were asked to name a maximum of ten anaesthetists who they believe had the necessary skills and abilities to perform at the highest level of their discipline. Respondents were asked to focus on the perceived expertise of the physician, rather than on sympathy or friendship with the person. The frequency distribution of votes determined the list of experts. The sample size of experts was determined a priori to equal the number of novices. As a result of the limited number of trainees, data saturation did not determine sample size. Following the evaluation of the peer assessment the first investigator explained the background and intention of the interview study to the nine experts chosen and asked them to participate.

### Setting

The setting of the data collection was identical for all interviews. Interviews were held at the end of a working day in a breakroom at the first author’s department. No other person was present during the interview besides the participant and the researcher.

### Data collection and analysis

After answering participants’ questions about the study and obtaining written informed consent, the investigator engaged in a 25 to 55-min semi-structured interview with each participant. Semi-structured interviews included specific questions, but they were flexibly conducted so that participants were free to elaborate or discuss associated topics. Basic themes concerning “expertise” were identified from the literature review prior to data collection. Additional themes emerged during the interviews. The questions for the novices and for the experts (Table 1) had extensive conceptual overlap and at the same time allowed for the difference in clinical experience. No pilot testing of the interview questions was done. All interviews were audio-recorded using a recording app on the iPad and stored offline as mp3-files for transcription and further analysis. Audiofiles were transcribed using ‘f5transcript’ (dr.dresing & pehl GmbH) and the resulting *.rtf-files were imported together with the *.mp3-files into ‘f4analyse’ (dr.dresing & pehl GmbH; www.audiotranskription.de) to create codes, write memos and summaries, and to export quotations. Transcription of audio files was started at the beginning of October, after all interviews had been conducted. Due to a time lag, transcripts were not returned to participants for comment or correction so participants did not provide feedback on the findings. No repeat interviews were carried out. Field notes were made during the interviews and imported as memos into ‘f4analyse’.

**Table 1:** Leading questions for the semi-structured interview with novice and expert anaesthetists. (translated from German)

Questions for the novice	Questions for the expert	Scope of the question
What was your motivation to become an anesthesiologist?	What was your motivation to become an anaesthetist?	Open-ended question introducing the interviewee to the topic.
What fascinates you most about this profession?	What fascinates you most about this profession?	Open-ended question introducing the interviewee to the topic.
Which tasks are part of being an anesthesiologist?	Which tasks are part of being an anesthesiologist?	Scope and task requirements of an anesthesiologist.
How did you experience your first months as novice anaesthetist?	What do you remember about your time as novice anaesthetist?	Personal account of being/having been a novice anaesthetist
Imagine that we meet ten years from now. What do you think will have changed by then?	Meanwhile, you have become an expert anesthesiologist. What changed in these years? Which factors are responsible for this change?	Paraphrase of expertise, description of being expert
What do you think will need to happen for this change to occur?	What has made you the person you have become?	Personal account of developing expertise. What can a person actively contribute to this development and what ‘just happens’?
	If a novice would ask you: “I would love to become such an expert anesthetist as you are; what should I do, read, and learn?” What would you answer that person?	Personal account of the professional development process. Recommendations on strategy to become an expert.
What will be your biggest challenges? How will you solve them?	Reflecting upon your personal journey of becoming an expert: What were your biggest challenges? How did you solve them?	Which problems are anticipated/were experienced in becoming an expert?
Can you remember a recent critical event where you had the impression that there is still so much to learn and that you are still at the very beginning of your professional career?	Can you remember your first months as anesthesia trainee? How did that feel like to be a novice? What were the challenges?	Perception of novice of how it feels to be a novice. Description of problem solving at novice stage
Tell me about that situation. How did that feel like?		
What role does medical textbook knowledge play and what role experience?	What role does medical textbook knowledge play and what role experience?	Concepts of explicit and tacit knowledge; interaction between these two knowledge representations
What role do ‘standards’ play? Is it possible or sometimes necessary to deviate from standards in order to ensure safe patient care? If yes, how do you decide?	What role do ‘standards’ play? Is it possible or sometimes necessary to deviate from standards in order to ensure safe patient care? If yes, how do you decide?	Concepts of variability, adaptation and the expert’s role in defining when to follow standards and when to deviate.
As a final question: how would you define “expertise in anesthesiology”?	As a final question: how would you define “expertise in anesthesiology”?	Assumption that after having been interviewed for 30–45 min on practical aspects of expertise, interviewees might be more able to give a theoretical definition.

All of the interviews were analyzed by one data coder (M.St.P) using Kuckartz’s approach for qualitative content analysis [20]. The objective in qualitative content analysis is to systematically transform a large amount of text in an iterative set of analyses and syntheses into a organized and concise summary of key results. This process of thematic analysis groups the data into themes and examines how the themes are interconnected within and across interviews [21, 22]. Coding was deductive where the major themes of the interview questions (Table 1) were used as coding themes and inductive where new themes emerged while reading the interviews. After the first pass through all the interviews the text passages of all categories were checked for conceptual overlap and categories merged and sometimes renamed. In a second pass, all interviews were re-analyzed with the shortened and condensed list of themes and categories and the coding was modified if necessary. In the final step, all text passages associated with a certain category were re-checked to be certain their meaning was adequately captured by the subtheme. Appendix I contains the final list with categories and their description (Appendix I).

### The researcher

All interviews were conducted and analysed by the same researcher (M. St.P) who at the time of the study was an attending anesthesiologist and had been working at the department for 26 years. He had basic training in qualitative research methodology as part of a masters program. The relationship of the researcher to the nine experts was collegial to friendly. Four of the nine novices were unknown to the interviewer prior to establishing first contact. In one case the interviewer had examined the resident (NOV_09) during her final medical exam 5 months prior to the interview. During the data collection period the interviewer kept a diary in which he wrote down field notes, personal reflections about his relation with interviewees, and how comments and statements from the interviews could inform his perspective in the subsequent days.

## Results

**Fig. 1:**
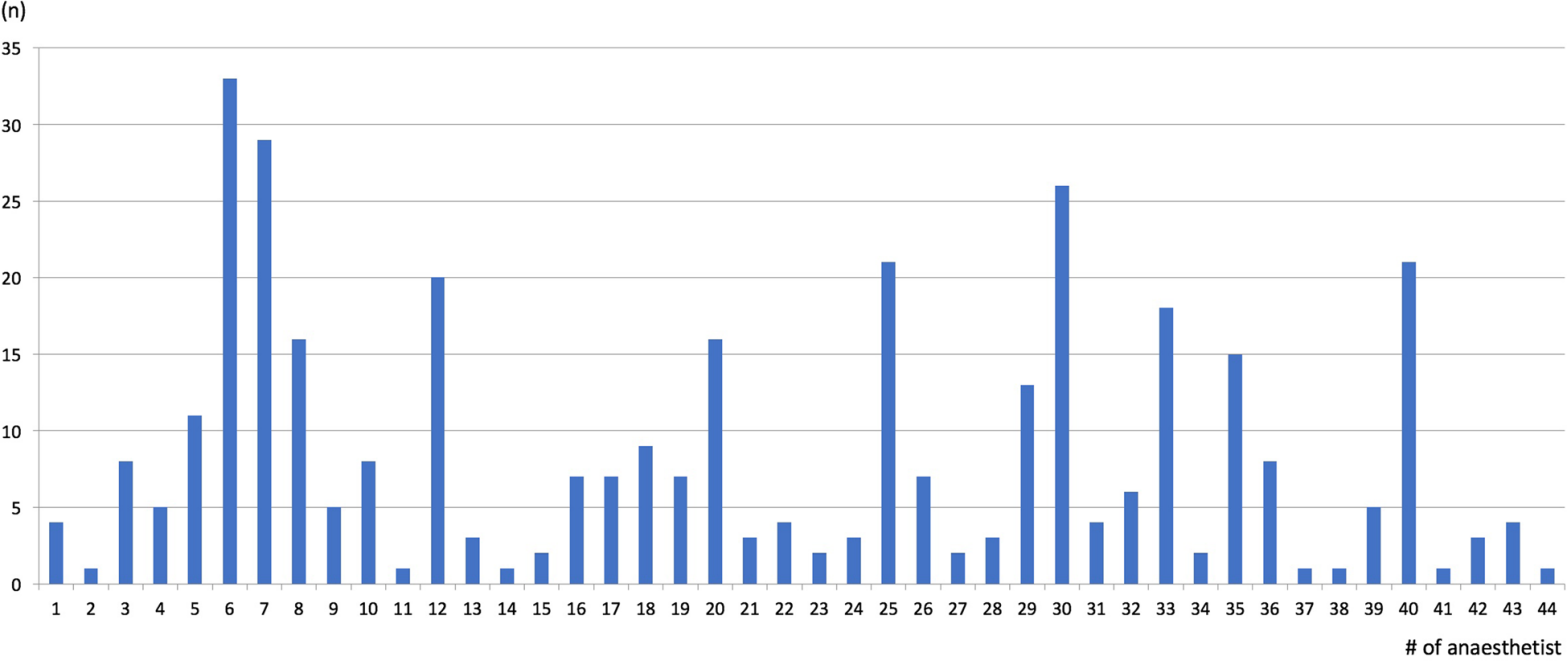
Peer assessment of experts. The ratings from 79 questionnaires resulted in a wide range of 44 anesthetists who were considered by their peers to be expert anaesthetists. Individuals with the nine highest ratings were included in the expert group

Nine trainees fulfilled the inclusion criteria and were asked to participate. Seventy-nine anaesthetic nurses and anesthesiologists returned their peer assessment of experts. The cumulative rating of the questionnaires returned resulted in a wide range of ratings with only two attendings standing out of the group (#6, #7; Fig. 1). The anaesthetists with the nine highest ratings were asked to participate in the interview study. No potential interviewee refused to participate or dropped out during the interview. Participants gave their written informed consent to the recording and offline-analysis of the recorded data, and anonymous use of quotes for publication. Anonymity and confidentiality of interview data was ensured by replacing the interviewee’s name with either ‘NOV_01 – NOV_09’ (for novices) or ‘EXP_01 – EXP_09’ (for experts).

The mean duration of clinical practice in anesthesia was 4.3 months for novices (range: 3–6 months) and 26.7 years for experts (range: 15–38 years). Two thirds of novices were women, whereas all of the experts were men. This sex ratio reflects the current gender balance at the department. The participant characteristics are summarized in Table 2.

**Table 2:** Participant characteristics and duration of interviews

Participant identification: novice	Sex	Months of anaesthesia practice	Duration of interview (min:sec)	Participant identification: expert	Sex	Years of anaesthesia practice	Duration of interview (min:sec)
NOV_01	F	6 mo	56:12	EXP_01	M	30 yrs	51:03
NOV_02	F	3 mo	25:19	EXP_02	M	29 yrs	43:21
NOV_03	F	4 mo	37:17	EXP_03	M	38 yrs	35:05
NOV_04	F	3 mo	28:00	EXP_04	M	35 yrs	41:08
NOV_05	F	5 mo	28:02	EXP_05	M	37 yrs	36:04
NOV_06	M	5 mo	39:14	EXP_06	M	15 yrs	39:03
NOV_07	M	4 mo	38:18	EXP_07	M	15 yrs	55:00
NOV_08	F	3 mo	32:58	EXP_08	M	23 yrs	42:55
NOV_09	M	6 mo	35:17	EXP_09	M	19 yrs	48:18

Interviews with novices were shorter than interviews with experts (novices: mean duration 35:27 min; range 25:19–56:12 min) vs. experts: (mean duration 43:33 min; range 35:05–51:03 min).

The following subheadings reflect the most relevant categories of the content analysis (appendix 1) together along with some representative quotations from interviewees.

### Complexity

One of the major challenges novices faced during the first months was the perceived complexity of their new work environment. They often described feeling being overwhelmed by what was routine work. Although experts recalled strong emotions from that time (e.g., anxiety, being thrown into the deep) they did not mention complexity as a major problem. Instead, they talked about the stress of the first months in rather general terms.

### Mentoring

All trainees reported that they felt secure and well supervised, that calling for help was actually expected, and that help was always available when needed. In contrast, not all experts had experienced such systematic mentoring and good supervision during their foundational training.

*„At the beginning of your career, they simply threw you into the deep. I only had a short familiarization period (...) and all of us were basically autodidacts.” (EXP_03)*

Novices and experts both shared the understanding that the development of expertise is not merely an individualistic endeavor but also a socially embedded process that is dependent upon peers willing to share their experiences with others.

*„Of course, you can’t achieve that all by yourself. Obviously not, you have many colleagues who support you in this development with a constant stream of new ideas, new suggestions, or discussion that you have. How do you do that, for example. As a result, it is not an independent learning process but rather a collective one where you make progress along with your colleagues.” (EXP_02)*

### Passive and active process

Most novices had not yet thought about the question of expertise in part because their work days were still filled with a relentless stream of new impressions, tasks and experiences. Those who did comment on the development of expertise assumed that becoming an expert was more or less a passive process - one which largely just happens over time.

*„It is an ongoing process; I really believe that it will happen over the years. And that it will come naturally as a result of continuously working in the operating theater.“ (NOV_02)*

When asked about active components of expertise development, novices most often mentioned reading textbooks and attending educational events, with only one novice addressing the aspect of ‘leaving your comfort zone’. By contrast, leaving one’s comfort zone, investing additional time, and deliberately taking on challenges were central to the understanding of experts of what it needs to make progress and to become an expert.

*„I believe that if you always want to stay within your comfort zone, you will never acquire this broad range of experience and, in addition, you gain self-confidence and trust in your abilities with every difficult situation you were able to master successfully.“ (EXP_04*)

### Standards and guidelines

Novices and experts agreed that standards provided a valuable introduction to current, safe anaesthetic practice. Several novices assumed that written standards were specific enough to actually address every clinical contingency and that following written procedures would guarantee patient safety. In contrast, experts stated that they had learned to negotiate experience and written guidance and that standards sometimes had to be put aside for the sake of safe patient care.

*„In this type of situation, the guidelines would recommend (...) and you say: Yes, BUT! And you have to be capable to justify this ‘but’, that’s the crucial point.“ (EXP_05)*

*„It could be that you don’t meet the standard, but you may more than meet the requirements of the situation.“ (EXP_07)*

### Quasi-normative rules

Several novices mentioned that the real challenge was less to familiarise oneself with the written standard operating procedures (SOP’s) at the department, than to learn the unwritten, quasi-normative rules of each of his/her supervising senior consultant [23].

*„In daily practice especially as novice you sometimes orientate yourself very much on what the consultant might want you to do in a certain situation, even if you have to acknowledge that the guidelines you read tell you otherwise and that the next consultant will want you to do things in yet another way.“ (NOV_07)*

It was left for novices to puzzle out the reason for discrepancies between the rules of one senior staff member or another. It was also left to novices to learn how to decide which of these rules he or she should apply given the patient and which he might want to apply himself in the future..

### Decision making

Novices described decision making as a very rational, conscious, and deliberate step-by-step process. Their responses contained no references to anything like tacit knowledge or intuitive decision making.

*„I sometimes imagine that an expert carries a very extensive flowchart in his mind and knows: if this, than that and if this and that, than preferably this option.“ (NOV_08)*

For experts, in contrast, the aspect of a holistic situational assessment and of an intuitive decision making was central to their understanding of how their decision making had evolved over the years. Terms used by experts were ‘gut feeling’, ‘7th sense’, and ‘intuition’.

### Limitations of expertise

During the brief introduction to the aims of the questionnaire 71 respondents (89%) asked the author for further clarification about what kind of expert he was looking for. The general understanding by the respondents was that there was no such thing as “*the* expert in anesthesiology” but rather that an anaesthetist can only be considered expert in a particular subspecialty. The understanding that expertise was limited to a one area of work (e.g. to pediatric anaesthesia, to regional anaesthetic techniques etc.) was shared by experts but not mentioned by novices. Experts also stated self-critically that expertise had a shelf life and that experts had to continue to use their skills or else would lose their expertise.

„*I do think that it’s part of expertise that you have to stay on the ball. I can hardly imagine expertise as something where you say: Well, for the next couple of years I will not concern myself with this topic; so yes, I do believe that expertise has an expiratory date.“ (EXP_01)*

### Teamwork

For experts expertise in anaesthesia cannot be confined to the individual acts but can also is manifested in the interaction with others. Over the years, what is considered as expertise has extended to things like working together as a team and working with members of other specialties and professional groups.

*„And finally, one component that I had completely undervalued at the beginning is ( … ) teamwork. You only can manage complex emergencies if you work as a team because you need the expertise of the others as well, neonatology, cardiology and so on.“ (EXP_01)*

## Discussion

In this study, the perspectives of nine clinical trainees from a German university hospital on expertise in anaesthesiology were contrasted with the viewpoints of nine expert anaesthetists. At the time of the interview trainees described their first months of residency training as a very positive experience. The level of supervision and systematic mentoring received from more senior residents and from the attending physicians enabled novices to perform a broad range of unfamiliar activities in a safe learning environment. We do not take this feedback as granted as previous studies have reported a lack of adequate supervision in many medical specialties during the first months of residency [24, 25]. The discrepancy between what novices experienced and what experts were able to remember from their first months as novices may not only be the result of mere forgetfulness by experts but rather of the preponderance of using readily available knowledge about current performance over their own former learning experience [26]. As people become more expert, they automate manual or simple tasks and develop a view of the task at hand in which the details of the task become less salient. As a result, expert supervisors may have difficulty understanding the particular kinds of challenges faced by novices when trying to familiarise with a new clinical environment and to learn novel tasks, even when reminded of these challenges. The interviews were intended in part to answer the research question of what novices think ‘expertise’ might entail and how this could inform the expert’s approach to clinical teaching. We can conclude from the novices’ responses that they actually do not deal with the same problems as researchers when it comes to understanding expertise. Novices struggle with very basic issues like translating theoretical knowledge into meaningful and appropriate action and acquiring vital skills. They do not spend much time thinking about expertise: it is simply not yet on the horizon. Their almost complete lack of concern in developing expertise is also reflected in the novices’ responses about what they can contribute to becoming an expert. The main issues they raised were the acquisition of theoretical and practical knowledge and the need for a supportive environment. Experts, by contrast, stated that seeking challenges and continuously stretching performance boundaries had been vital for becoming an expert. In a similar vein, the notion of leaving one’s comfort zone and of practicing deliberately have been identified as a hallmark of excellence [27, 28]. We interpret the relative absence of the idea of leaving one’s comfort zone from the novices’ responses as an indication that novices can only start leaving their comfort zone once they have found one, and this requires a solid basis of clinical experience.

A common understanding shared by every novice and expert was that the development of expertise was a socially embedded process facilitated by an organisation where peer discussions were possible without fear of being ridiculed and where mentors and peers were willing to share their knowledge. The social nature of expertise reappeared in the understanding of experts that expertise in anaesthesia emerged from teamwork endeavors, often on an interdisciplinary level with a range of clinical partners. Both groups agreed that every expertise will eventually reach its limits and that it might be a challenge for the expert to accept this and ask for help. In contrast to studies on help-seeking behavior in surgeons [29], where calling for help is seen as a threat to the expert’s image, autonomy, and development as independent practitioner was not mentioned by our experts. A characteristic feature of all the responses was the assessment that expertise was neither a monolithic feature, readily recognizable by everyone nor a quality that, once acquired, would necessarily endure over an entire professional life. Similar to other interviewees [18] our respondents believed that expertise depends to some extent on s social context and is in an important sense, never fully general [30].

While expert supervisors had difficulty understanding the particular kinds of challenges faced by novices during their first months, novices were unable to comprehend the dilemmas experts face when forced to reconcile strict guidance by evidence-based rules and personal experience in the treatment of an individual patient. Novices saw rules as the embodiment of the best possible way of carrying out activities, covering all known contingencies. They assumed that following written procedures would guarantee patient and staff safety. Expert anaesthetists, by contrast, took the view that rules and standards were essentially underspecified, requiring experience and expertise to translate them to any specific situation [31]. This is because the variability of diseases and patients and the interactions across patient conditions spill over the category boundaries of best-practice guidance. In addition, the scientific evidence presented in written guidelines and recommendations does not always speak for itself but needs to be interpreted, revised, and tailored to specific contexts and conditions, all of which takes experience and expertise [30, 32–34]. As a result, experts believed that in some situations patient safety could only be guaranteed by not following rules if this was supported by a valid mental model or social understanding or both of the situation [35]. Interestingly, these two perspectives mirror the two contrasting ways of thinking about the functions, strengths and limitations of rules and standards safety science knows of [31].

While experts commented on the challenges they encountered when trying to negotiate evidence-based medicine (EBM) standards and personal experience, trainees stated that that evidence-based standards often played a subordinate role to the ‘quasi-normative rules’ [23] consultants had established. To our knowledge, this aspect of expertise development has not yet been mentioned in the anaesthesia education literature. For experts, experience may occasionally trump EBM-based rules. For novices, it appears, quasi-normative rules always trump things like EBM-based rules.

A central premise of theoretical and empirical research about expertise is that it is the level of tacit knowledge acquired that distinguishes experts from novices. Given the fact that the novices had only recently finished medical school and had on average 4 months of clinical experience at the time of the interview, it does not come as a surprise that their responses did not reveal any understanding of tacit knowledge or intuitive decision making in anaesthesia. Instead, novices conceptualized decision making as a very rational, conscious, and deliberate step-by-step process. This observation may reflect the fact that a person often cannot imagine, let alone conceptualize an experience he or she has not yet had. Instead, similarity matching and representativeness favor current and familiar experiences as the basis for understanding [26] which in the case of recently graduated medical students most probably will be hypothetico-deductive reasoning taught and prioritized in higher education. Experts, in contrast, knew exactly what intuitive decision making feels like and were able, at least to some extent, to talk about it. In addition, they reported that they checked their intuitions with conscious deliberation before acting upon the first, an approach that has been termed ‘informed intuitions’ in the literature on decision making [32].

### What this study adds to our understanding

The current research adds three aspects to the medical education literature on education for expertise development: First, it describes the perspective of novices in their first months of clinical rotation on the development of expertise. To our knowledge, no previous qualitative research has explored junior residents’ perspective on this topic at this point in their careers. Although the data of this study was gathered in anaesthesiology there is reason to believe that novices in other specialties face similar challenges when trying to familiarise themselves with routine clinical work. Taking into account the viewpoint of novices can help expert clinical teachers to identify concepts shared by both groups and to focus their teaching on aspects of professional development novices are currently unaware of.

Second, the responses given by the experts illustrate the fact that neither clinical experience alone nor strictly following evidence-based measures are a sufficient prerequisite to create expertise. Rather, the successful negotiation of evidence derived from clinical research with experience and pathophysiological knowledge in the treatment for an individual patient is a pivotal moment in the development of expertise [34].

Finally, the novices’ comments on the challenge of how to familiarize themselves (and reconcile) the ‘quasi-normative rules’ [23] of different attendings draws attention to a hidden curriculum of residency training that could inhibit the development of mature clinical decision making. These quasi-normative rules may be an unwanted result of the traditional apprenticeship model of residency training in which trainees are expected to imitate the role model of the supervisor [36]. In a more resident-directed model experts should render the processes involved in their problem solving of complex cognitive tasks more explicit, hereby teaching the trainee how to articulate the reasoning behind a decision [37]. Rather than taking a normative stance towards the one right treatment path, expert supervisors should address the complexities, nuances, and ambiguities of clinical situations to enhance decision making skills among novices [11]. To strike the right balance between acceptance of ambiguity and plurality of clinical work on the one hand, and clear guidance of unexperienced novices on the other, is certainly not an easy task for clinical educators. Nevertheless, whenever expert supervisors expect residents to strictly follow ‘quasi-normative rules’ despite the danger that such rules can oversimplify [38] they may actually delay the development of critical clinical thinking, an ability that residency training programs in any specialty should be designed to encourage [39].

### Limitations

The limitations to this study are identical to those of any qualitative interview study based on a small number of participants [40–42]. The interview was performed at one location and data has been collected from employees at the Department of Anesthesiology at one German University hospital. Therefore, the responses of the participants reflect the organizational culture and structure of one hospital. It follows that the generalizability and transferability of the study results to other contexts will be limited. The convenience sample size of 9 novices and 9 attendings may not have allowed for data saturation and increasing the number of interviews might have strengthened the results of the study.

Further, the investigator was a colleague and in the case of the novices a direct supervisor which may have affected the responses in a negative or positive way. That is junior participants may have felt compelled to respond in a more socially acceptable fashion rather than feel free to provide a true reflection of their opinions. On the other hand, as the interviewer has worked with many of the experts, it is possible that this may affected the openness of the verbal exchange. Finally, the data was analysed and coded by only one investigator (M.St.P) which might have introduced a systematic bias into the research.

## Conclusions

Despite these limitations we are confident that we were able to identify important key themes relevant to the different perceptions of novices and experts on the development of expertise. Although the data was gathered in anaesthesiology, our results may help to sensitise the expert clinical teacher from any medical specialty to understand and build on novices’ perspectives on what they think they will have to learn, see, and do in order to develop expertise. Integrating the trainees’ perspectives into postgraduate training may help create an educational alliance [14] and help taylor the syllabus and teaching efforts during the months of clinical apprenticeship more to the needs of the novices. In our opinion, the viewpoint of novices can help expert clinical teachers to identify concepts shared by both groups to build upon and to focus their teaching on aspects of professional development novices are currently unaware of.

Although the explicit aim of our training efforts is to help novices integrate and reconcile different types of knowledge, clinical educators should be aware of the danger that novices are taught instead to prioritize the ‘quasi-normative rules’ of different consultants over critical thinking and the negotiation of evidence-based guidelines with clinical experience. Not acknowledging and addressing these hidden agendas in residency training could actually delay the development of good clinical judgment and proficiency in anaesthesia as well as in any other medical specialty.

Based upon the findings of this study clinical educators are recommended to share with novices how they negotiate evidence derived from clinical research with their clinical experience and with pathophysiological knowledge. The opportunity to learn from experts how they break through the barriers of strict guidance and reconcile diverse strands of knowledge in the treatment for an individual patient might create precious learning moments for novices and help them progress towards expertise in their respective medical specialty.

## Data Availability

The datasets used and/or analysed during the current study are available from the corresponding author on reasonable request.

## Appendix

**Table 3:** List with categories and their respective occurrence in the interview data of novices and experts

Theme	Category	Description	Mentioned by novices	Mentioned by experts
**Being a novice**	*Learning the basics*	Account of learning experiences during the first months	x	x
	*Complexity*	Descriptions of clinical situations in combination with feelings of being overwhelmed, difficulty understanding the situation	x	
	*Mentoring*	Mentors and clinical teachers, social support	x	x
	*Quasi-normative rules*	Following the consultant’s rules, anticipating what the superior’s rule might be	x	
	*Patient safety*	Reference to patient safety	x	x
	*Making progress*	Descriptions of personal progress over time	x	
	*Carrying responsibility*	Being responsible for a patient; the weight of responsibility	x	
	*Learning*	Learning strategies, learning from experience	x	x
	*Negative aspects*	Account of difficult circumstances, difficult situations during the time as novice	x	x
**Being an expert**	*Goals*	Into which kind of clinician does the novice want to evolve	x	x
	*Passive process*	Reference to passive aspects of becoming an expert	x	
	*Active process*	Reference to active aspects of becoming an expert	x	x
	*Social support*	Reference to peers supporting the novices’ development	x	x
	*Challenges*	Challenges and difficulties on the way to becoming an expert	x	x
**Knowledge and experience**	*Textbook knowledge*	References to explicit, textbook knowledge	x	x
	*Experiential knowledge*	References to implicit, experiential knowledge	x	x
	*Knowledge and experience*	Relationship between textbook knowledge and experience	X	X
	*Standards and guidelines*	References to standards and guidelines, assessment of the role standards and guidelines play	x	x
	*Violating standards*	Not following rules in order to create patient safety	x	x
	*Being lucky*	Reference to luck as a third pillar besides knowledge and experience		x
	*Decision making*	Descriptions of how an expert makes decisions	x	x
**Defining the expert**	*Difficulty with*	Difficulty or inability to define “expert in anaesthesia”	x	
	*Board certification*	Reference to the board certification and its relationship to expertise	x	x
	*Dexterity*	Manual dexterity	x	x
	*Crisis management skills*	Crisis management as core competence of the anaesthetist	x	x
	*Risk assessment*	Reference to assessing risk in patients		x
	*Intuition*	Mention of ‘intuition’, ‘7th sense’, ‘gut feeling’		x
	*Limitations of expertise*	Negative aspects of expertise		x
	*Teamwork*	Reference to the social/teamwork component of expertise		x
	*Nature or nurture*	Interaction of personal characteristics and contextual factors in the formation of an expert	x	x

## Abbreviations

EBM: Evidence Based Medicine
SOP: Standard Operating Procedure
